# Change in the family food environment is associated with positive dietary change in children

**DOI:** 10.1186/1479-5868-10-4

**Published:** 2013-01-07

**Authors:** Gilly Hendrie, Gundeep Sohonpal, Kylie Lange, Rebecca Golley

**Affiliations:** 1 Commonwealth Scientific Industrial Research Organisation (CSIRO) Food and Nutritional Sciences, Adelaide, Australia; 2 Department of Nutrition and Dietetics, School of Medicine, Faculty of Health Sciences, Flinders University, Adelaide, Australia; 3 Centre of Clinical Research Excellence (CCRE) in Nutritional Physiology, Interventions and Outcomes, Discipline of Medicine, The University of Adelaide, Adelaide, Australia; 4 Public Health, Sansom Institute for Health Research, University of South Australia, Adelaide, Australia

**Keywords:** Children, Family food environment, Feeding practices, Behaviour change

## Abstract

**Background:**

The family food environment is an important influence in the development of children’s dietary habits. Research suggests that influences of current dietary behaviour and behaviour change may differ. The aims of this paper were to: (1) investigate the association between the food environment at baseline and change in children’s saturated fat intake; and (2) to explore whether a change in the food environment was associated with a change in children’s saturated fat intake.

**Method:**

Secondary analysis of a 12 week cluster randomised controlled trial in 133 4-13 year old children. Families were randomly allocated to parental education regarding changing to reduced-fat dairy foods or a comparison non-dietary behaviour. The interventions were family focused. Parents received education from a dietitian in 3x30minute sessions to facilitate behaviour change. Parents completed a comprehensive questionnaire capturing three domains of the food environment – Parent knowledge and attitudes; shaping practices; and behaviours and role modelling. Children’s dietary intake was assessed via multiple 24-hour recalls at baseline and week 12. Changes in the family food environment and primary outcome (saturated fat) were calculated. Hierarchical linear regression models were performed to explore the association between baseline and change in food environment constructs and change in saturated fat intake. Standardised Beta are presented (p<0.05).

**Results:**

After adjustments for child and family demographics, higher levels of perceived food availability (β=-0.2) at baseline was associated with greater reduction in saturated fat intake, where as higher perceived responsibility (β=0.2), restriction (β=0.3) and pressure to eat (β=0.3) were associated with lesser change in saturated fat. An increase in nutrition knowledge (β=-0.2), perceived responsibility (β=-0.3) and restriction (β=-0.3) from baseline to week 12 were associated with greater reduction in saturated fat intake.

**Conclusions:**

The present study was one of the first to quantify changes in the family food environment, and identify a number of factors which were associated with a positive dietary change. Because interventions focus on behaviour change, the findings may provide specific targets for intervention strategies in the future.

**Trial registration:**

Australia New Zealand Clinical Trials Registry ACTRN12609000453280.

## Introduction

Children’s diet quality is poor with implications for health in childhood [1-3] and later life [4]. The Social Ecological Model considers the context in which a person is located, and for children the family has been highlighted as one of the important influences of health [5]. Characteristics within the family that influence or shape children’s dietary behaviour have been referred to as the family food environment [6].

The family food environment is a complex domain, and is thought to include parental factors such as nutrition knowledge, their parenting styles and feeding practices, role modelling, food availability and accessibility, as well as children’s own individual characteristics and behaviours [5]. Over the last decade an expanding number of studies have shown aspects of the family food environment to have strong modifying effects on children’s dietary behaviours. For example, higher maternal nutrition knowledge has been associated with healthier diets and lower body weight in children [7,8]. Parent feeding practices such as restriction and monitoring of children’s intake have been shown to influence children’s diet [9-11]. Greater parental pressure to eat has been associated with higher energy and fat intake, and increased consumption of energy dense foods [6,12,13]. In terms of the broader family environment, food availability and role modelling of eating behaviours have also been identified as important influences [6,13-16]. Parents can model positive, healthy behaviours such as consuming fruits and vegetables [13], or less healthy behaviours such as consuming sweet and savoury snacks [17].

Although the current literature suggests the family food environment is important, the majority of this evidence has come from cross-sectional research. Longitudinal research and interventions can provide stronger evidence of cause and effect. Interventions change behaviour by changing mediating variables, and interventions are more likely to be effective if the variables are strongly related to the behaviour [18]. Given that the family food environment is closely related to children’s behaviour, we need to understand whether changes in the family food environment are associated with change in diet.

A small number of longitudinal studies have examined the family environment in general but fewer have focused on the food environment. One Australian study of adolescents found mother’s modelling of healthy eating, and support from the family and best friends were associated with positive changes in fruit and vegetable intakes [19]. A second Australian study, of younger children, found the food environment determinants associated with weight status in the cross-sectional analysis were different to those associated with children’s weight in longitudinal analysis [20]. Factors associated with current dietary behaviour may also be different to the predictors of change in dietary behaviour. This is of relevance to the design of interventions because behaviour change is the primary objective of nutrition interventions.

This paper utilises data from a longitudinal randomised controlled trial examining the effects of parental behavioural nutrition education to encourage a change in children’s dairy food choices from regular to reduced fat varieties. The intervention was based on the family food environment model [5]. Parents were provided with information to increase their nutrition knowledge and equip them with the skills to better enable them to change their children’s diets. They were also encouraged to support dietary change through increasing the availability of reduced fat dairy products at home, encouraging the use of reduced fat products and role modelling the consumption of these products. We have reported elsewhere that the intervention was successful in changing dairy food choices and reducing children’s saturated fat intakes by 3.3 percentage points [21]. Here, we are interested in whether aspects of the family food environment were associated with this change in saturated fat. Therefore, the aims of this paper were to: (1) investigate the association between the food environment at baseline and change in children’s saturated fat intake; and (2) to explore whether changes in the food environment were associated with change in children’s saturated fat intake.

## Method

### Intervention, participants and recruitment

The study was approved by the Commonwealth Scientific Industrial Research Organisation (CSIRO) Food and Nutritional Sciences Human Ethics committee, was registered in the Australia New Zealand Clinical Trials Registry (ACTRN12609000453280), and the method is described in detail elsewhere [21]. In brief, families included in this secondary analysis were those in the intervention or control group who completed the main study. The families were parents with at least one child (aged 4-13 years), recruited using public advertisement, and a CSIRO participant database. Children were usual consumers of regular fat dairy foods (defined as the majority of milk, cheese, yoghurt, icecream or custard was regular fat). Parents were asked to report their children’s usual dairy consumption, by fat type, during screening. Families were randomised to one of two groups. The intervention group received advice regarding a switch from regular fat to reduced fat dairy foods, from a dietitian during 3x30 minute face-to-face sessions held one month apart over 12 weeks. The intervention provided information about the importance of dairy foods, the fat content of various dairy foods and current recommendations for children, common barriers to consumption were addressed, and parents were provided with a comprehensive pictorial shopping guide to assist with dairy food purchases. Information was provided in a standard intervention booklet.

The comparison intervention was also family based; however, it focused on reducing children’s screen time behaviours (with the aim of maintaining current diet). Families also received a standard intervention booklet and equal contact with the dietitian.

### Outcome measure: dietary intake

Children’s dietary intake was measured at baseline and end of intervention (week 12) via three 24-hour diet recalls. The recalls covered two weekdays and one weekend day, and one was conducted face-to-face, the other two via telephone. The three pass recall is a well established method and is described in detail elsewhere [21]. Parents were the primary source of recall for children aged 10 years or less, with clarification sought from the child where appropriate (for example for additional foods consumed at school). Children aged 11-13 years were the primary source of their own recall, with assistance from parents when required (for example for brand names or recipes). Families were provided with a Food Model Booklet, adapted from the US Department of Agriculture [22], showing life sized pictures of common household measures to assist in the estimation of portion size. Information was entered by a research dietitian into Foodworks Professional (Xyris Software Pty Ltd, Queensland, Australia) to allow nutrient and food group estimation. Nutrient and energy intakes were estimated using Australian food composition data [23].

The primary outcome was change in percentage of energy from saturated fat. Grams of saturated fat intake was converted to kilojoules (multiplied by 37 kilojoules/gram), and the percent contribution to total daily energy intake calculated. The average percentage of energy from saturated fat over the three recalled days was calculated.

### Exposure variables: family food environment

At baseline and at the end of the intervention parents completed a questionnaire encompassing a broad range of aspects of the family food environment.

### Parents’ Nutrition knowledge

Nutrition knowledge was measured using the General Nutrition Knowledge Questionnaire [24], validated for use in Australian adults [25]. The questionnaire covered four areas of general healthy eating and nutrition knowledge – knowledge of nutrition recommendations (13 items), food sources of nutrients (70 items), choosing ‘everyday’ foods (10 items) and diet-disease relationships (20 items). Correct answers (1 point each) were summed, giving a total possible score out of 113.

Three additional questions specific to dairy were also asked: “What type dairy foods do experts say children over the age of 2 years should eat?”, “The *Dietary Guidelines for Children and Adolescents* suggest children should consume how many serves of dairy foods each day?”, “Reduced fat milk contains less fat than low fat milk.”. One point was assigned for each correct answer (out of a possible 3).

### Child feeding practices

Five factors from the validated, and commonly used, Child Feeding Questionnaire (CFQ) [10] were measured: parents’ perceived responsibility for children’s food intake (3 items), concern for child weight (3 items), parent restriction (8 items), monitoring (3 items) and pressure to eat (4 items).

### Food environment

The family food environment was captured by combining questions from three different questionnaires. Questions from the Family Food Environment (FFE) questionnaire [26] were used to assess: perceived fresh food availability (7 items), perceived adequacy of child’s diet (7 items), meal preparation views (5 items), role modelling of eating behaviours (5 items), family involvement in meal preparations (4 items) and television interruptions to meals (2 items). Parents general level of food involvement was measured using the Food Involvement Scale [27], which has been shown to fit well with other measures of the family food environment [8]. Finally, the Comprehensive Feeding Practices Questionnaire (CFPQ) [28] includes aspects of parent feeding practices not captured by other questionnaires. Two constructs from this questionnaire were included – encouraging balance and variety in food intake (4 items) and teaching about nutrition using explicit techniques to encourage healthy foods (3 items).

This was the first time questions about the family food environment from these different sources have been combined, therefore exploratory factor analysis was used to establish the validity of the family food environment factors in this sample. The data was assessed and supported the use of factor analysis. Principal axis factor analysis revealed seven factors with Eigen values exceeding 1, but an inspection of the Screeplot revealed a six factor solution. Varimax rotation was performed to aid the interpretation, and the rotated solution is discussed. For all questionnaires, negatively worded questions loaded negatively, and were reverse coded in the creation of factor scores. Factor scores were created by calculating the mean score for the items loading onto each factor (in most cases out of a possible 5). The six factors of the family food environment were perceived responsibility, perceived fresh food availability, perceived adequacy of child’s diet, meal preparation views, food involvement in meal preparation, role modelling of eating behaviours, and TV interruptions to meals. The factors structures were generally similar to the original questionnaires [6,8]. Factor analysis of the two constructs from the CFPQ showed the original factors, encouraging balance and variety in food intake and teaching about nutrition, loaded onto one factor (one item “I tell my child what to eat and what not to eat without explanation” had a factor loading <0.35 leaving a 6 item factor), and in this paper was labelled as ‘teaching and encouraging child about food’. Four items (three from perceived food availability and one from perceived adequacy of child’s diet factor) had a factor loading of less than 0.35 and did not load onto the original factors. These were excluded from the analysis. The reliability of all factors showed acceptable reliability. A value of 0.7 or above is generally considered acceptable [29] (Cronbach’s alpha values presented in Table 1).

**Table 1:** Baseline and change scores for parental and family covariates and child outcomes

**Family food environment variables**	**Maximum possible score**	**Cronbach alpha (α)**	**Baseline**^**1**^		**Change**^**2**^				
			**Mean**	**SD**	**Mean**	**SD**	**Min**	**Max**	**Effect Size**^**3**^
Model 2: Knowledge and attitudes									
Dairy messages	3		1.3	0.9	0.5	1.1	-2.0	3.0	0.43
Nutrition knowledge	113	0.81	75	12	3	8	-29	21	0.33
Perceived responsibility	5	0.94	4.2	0.9	0.0	0.8	-2.0	4.0	0.00
Perceived fresh food availability	5	0.81	4.2	0.6	-0.1	0.6	-1.5	2.0	-0.09
Perceived adequacy of child's diet	5	0.93	3.9	0.8	0.0	0.6	-1.0	4.0	0.08
Parent’s meal preparation views	5	0.78	3.8	0.6	-0.1	0.6	-2.4	1.2	-0.16
Concern for weight	5	0.87	2.3	1.2	-0.1	0.9	-3.0	4.0	-0.13
Model 3: Parent shaping practices									
Teaching and encouraging child about food	5	0.80	4.2	0.4	0.0	0.4	-0.8	1.2	-0.06
Restriction	5	0.81	3.4	0.9	0.1	0.8	-1.5	3.8	0.07
Pressure to eat	5	0.72	2.5	1.0	-0.2	0.8	-2.8	2.3	-0.20
Monitoring	5	0.96	4.0	0.9	-0.1	0.7	-2.0	2.3	-0.14
Model 4: Parent behaviours and role modelling									
Food involvement	7	0.62	5.0	0.6	0.0	0.7	-2.7	3.5	0.05
Family involvement in meal preparation	5	0.60	2.6	0.6	-0.1	0.4	-1.3	1.0	-0.19
Role modelling eating behaviours	5	0.83	4.1	0.7	0.0	0.4	-0.8	1.6	-0.03
TV interruptions to meals	5	0.87	2.7	1.4	-0.2	0.7	-3.5	2.0	-0.24
Energy (kJ)			8040	1721	-256	1573	-3985	5158	-0.16
Saturated fat (% of energy)			15.3	2.7	-2.1	3.7	-13	5	-0.57

^1^ Baseline differences between treatment groups was assessed using Independent t test. *Significance p<0.05.

^2^ Change=follow-up minus baseline. Therefore a positive change value reflects an increase from baseline to follow-up.

^3^ Effect size estimate = mean change/change in SD [30].

### Covariables

#### Anthropometry

Height and weight of children and parents/caregivers was measured. Participants were weighed (model AMZ14; Mercury Digital Scales, Tokyo, Japan) and height measured with a stadiometer (Seca, Hamburg, Germany) while lightly clothed, without shoes, using a protocol consistent with international standards [31]. Height and weight was used to calculate body mass index (BMI: weight in kg/height in m^2^). Parent/caregiver weight status was classified using the World Health Organization definition, with BMI ≥25 kg/m^2^ overweight and ≥30 kg/m^2^ obese [32]. For children, BMI was converted to a z score, adjusted for age and gender using the least mean squares method [33]. Given the lack of Australian data, calculations were based on British reference data provided as a computer program [33]. Children’s BMI z score was classified using the International Obesity Task Force definition [34].

### Family questionnaire

Family demographic information was collected at baseline. Details were sought about parents’ gender, highest level of education, employment status, estimated annual household income. Child sex and date of birth were also reported.

### Statistical analysis

Differences in the baseline family food environment factors between groups was assessed using Independent Samples T-tests (significance p<0.05). Change scores were calculated for the family food environment variables and percent energy as saturated fat (change=Week 12 follow-up minus baseline). The effect size estimate of the change in family environment and dietary outcomes was calculated (mean change/change in SD). Values of <0.1 were considered small, 0.3 medium, and >0.5 large were used for interpretation of effect size [35]. The dependent variable was change in saturated fat, and a negative score reflected a (desirable) decrease from baseline. Saturated fat was chosen because regular fat dairy is a major source of saturated fat in children’s diet [36]. This intervention targeted a switch to reduced fat dairy as a means of reducing children’s saturated fat intake.

The independent predictor variables were baseline family food environment factors for Model 2-4a, and the change in family food environment factors for Models 2-4b. A positive score reflected an increase in factor scores between baseline and follow-up. Socio-demographic covariables were selected *a priori* based on known associations with both predictor and outcome (i.e. family food environment constructs and dietary intake). These included child gender, age, BMI z score, household income, caregiver education and BMI. The relationships between each of household income (4 categories) and education (4 categories: primary school or less, some/completed high school, vocational/trade qualification, tertiary degree) and saturated fat were explored and both showed linearity. Therefore, for regression analysis these were treated as continuous variables to reduce the size of the model. All variables were considered normally distributed.

Hierarchical linear regression models were performed to explore the association between socio-demographic variables, baseline and change in family food environment constructs and change in saturated fat intake. As the aim of the analysis was to consider the associations between family food environment and change in children’s percentage of energy from saturated fat, regardless of the mode of change, data from the intervention and control groups were pooled, and treatment was not included in the analysis.

Due to sample size limitations, three domains of the family food environment were explored in separate models. Inclusion of factors into these models was theoretically derived [5,37]. Model 1a included baseline socio-demographic variables. Model 2a and 2b Knowledge and Attitudes: included parent nutrition knowledge and dairy messages, perceived responsibility and concern for weight (from the CFQ), perceived adequacy of child’s diet, perceived fresh food availability and parent’s meal preparation views (from the FFE). Model 3a and 3b Parent Shaping Practices: included restriction, pressure to eat and monitoring (from the CFQ), and teaching and encouraging about food (from the CFPQ). Model 4a and 4b Parent Behaviours and Role Modelling: included food involvement, family involvement in meal preparation, role modelling of eating behaviours, and TV interruptions to meals (from the FFE). The family food environment factors were entered in step 1 (either baseline or change scores) with child (age and BMI z score) and parent characteristics (parent education, employment and household income) entered as the second step. Results are presented for the final adjusted models. Normality, linearity and homoscedasity assumptions were assessed and met. Results were considered significant when p<0.05. All analyses were conducted using SPSS 18.0 (2010, IBM Inc, USA).

## Results

Data analysed were based on 133 children from 86 families, representing 91% of the study sample. Primary caregivers were majority female (87%), 48% were within the normal weight range, and 29% were overweight. Fifty-six percent were tertiary educated, 44% worked part time and 54% reported a household income between A$52,400-114,399. The average child age was 8.9 (SD=2.9) years, 59% were male and 68% of the sample were categorised within the normal weight status category (Table 2). At baseline the mean percent energy derived from saturated fat was 15.3 % (Table 1).

**Table 2:** Sample characteristics based on 86 parents/caregivers of 133 children

**Parent characteristics**		**Frequency (percent) *unless otherwise stated***
Highest level of education	Some high school/ completed high school	20 (23)
	Technical or trade qualification	18 (21)
	Tertiary degree	48 (56)
Employment status	Full time	17 (20)
	Part time	38 (44)
	Homemaker	21 (24)
	Student	6 (7)
	Unemployed/retired/disabled	4 (5)
Estimated household income	A$20800-36300	14(16)
	A$36400-51999	11 (13)
	A$52400-77999	23 (27)
	A$78000-114399	23 (27)
	A$114000+	15 (17)
Weight status	Underweight	2 (2)
	Normal weight	41 (48)
	Overweight	25 (29)
	Obese	18 (21)
**Child characteristics**		
	*Gender*	79 (59) Boys 54 (40) Girls
	*Age (y) (mean(SD))*	8.9 (2.9)
	*BMI z score*^*1*^*(mean(SD))*	0.24 (1.16)
Weight status^1^	Underweight	14 (10)
	Normal weight	90 (68)
	Overweight	22 (17)
	Obese	7 (5)

^1^ BMI z scores calculated using British reference data and IOTF cutoffs [34].

Table 1 shows the mean general nutrition knowledge score was 75 (out of 113). At baseline, mean family food environment scores were highest (≥ to four) for six (out of 13) environment constructs – Perceived responsibility and fresh food availability (from Model 2 Knowledge and attitudes), Teaching and encouraging child about food and Monitoring (Model 3 Parent shaping practices), and Food involvement and Role modelling eating behaviour (from Model 4: Parent behaviours and role modelling). Table 1 also shows the changes in the family food environment construct scores, expressed as effect size. The effect size of the change in the family environment constructs varied between small (7 out of 15 family environment constructs), medium (6/15 constructs) and large (2 constructs) [35] (Table 1).

The average change in percent energy derived from saturated fat, from baseline to follow up, was -2.1% (range -13% to +5%; effect size -0.57). A negative value represents a decrease in saturated fat intake from baseline to follow up (Table 1).

The results of the regression analysis for baseline characteristics and change in percentage energy from saturated fat are presented in Table 3 (Model 1a). Child age (β=0.3, p=0.02) and BMI z score (β=-0.8, p=0.01) at baseline, but not child gender or family demographics, were associated with change in saturated fat (Table 3). Younger children reported the greatest reduction in percentage energy from saturated fat. By comparison, baseline BMI z score and change in saturated fat were inversely associated. Higher BMI z score was associated with a greater decrease in saturated fat between baseline and follow up (Table 3).

**Table 3:** Multiple linear hierarchical regression results for baseline family food environment characteristics and change in percentage of energy from saturated fat

	**β**^**1**^	**Std β**	**95% CI**		**P value**	**Adjusted R**^**2**^	**Model P value**
Model 1a Child characteristics						0.05	0.049
Child gender	0.4	0.1	-0.9	1.7	0.55		
Child age (years)	0.3	0.2	0.04	0.5	0.02		
Baseline BMI z score	-0.8	-0.3	-1.4	-0.2	0.01		
Household income^2^	-0.3	-0.1	-0.8	0.2	0.27		
Caregiver education^2^	0.1	0.01	-0.8	0.9	0.88		
Caregiver BMI (kg/m2)	-0.01	-0.02	-0.1	0.1	0.82		
Model 2a^3^ Knowledge and attitudes						0.12	0.006
Dairy messages (/3)	0.7	0.2	-0.1	1.4	0.07		
Nutrition knowledge (/113)	-0.0001	-0.0004	-0.1	0.1	0.10		
Perceived responsibility (/5)	0.8	0.2	0.1	1.5	0.03		
Perceived fresh food availability (/5)	-1.1	-0.2	-2.2	0.0	0.05		
Perceived adequacy of child's diet (/5)	-0.2	-0.04	-1.0	0.6	0.64		
Parent’s meal preparation views (/5)	-0.4	-0.1	-1.5	0.6	0.41		
Concern for weight (/5)	0.02	0.01	-0.6	0.6	0.95		
Model 3a^3^ Parent shaping practices						0.16	<0.001
Teaching encouraging child about food (/5)	-0.7	-0.1	-2.3	0.9	0.42		
Restriction (/5)	1.0	0.3	0.3	1.8	0.01		
Pressure to eat (/5)	0.9	0.3	0.3	1.6	0.01		
Monitoring (/5)	-0.7	-0.2	-1.6	0.1	0.08		
Model 4a^3^ Parent behaviours and role modelling						0.07	0.049
Food involvement (/7)	0.3	0.04	-0.8	1.3	0.61		
Family involvement in meal preparation (/5)	1.2	0.2	0.1	2.3	0.03		
Role modelling eating behaviours (/5)	0.7	0.1	-0.3	1.8	0.18		
TV interruptions to meals (/5)	0.1	0.1	-0.4	0.7	0.62		

^1^ Beta value represents the strength of the association with change in saturated fat (negative value for change is desirable). Therefore negative Beta means higher levels of the food environment construct were associated with greater reduction in saturated fat. Positive Beta means lower levels of the family food environment construct were associated with a greater change in saturated fat.

^2^ Highest level of education completed (4 categories: primary school or less, some/completed high school, vocational/trade qualification, tertiary degree) and total household income (4 categories) were treated as continuous variables.

^3^ Analyses account for child (gender, age and BMI z score) and family demographics (household income, parent education and BMI).

Two baseline knowledge and attitudes variables (Model 2a) were associated with change in saturated fat. Parent reported perceived responsibility for children’s food intake (β=0.8, p=0.03) at baseline was positively associated with change in saturated fat and perceived fresh food availability (β=-1.1, p=0.05) was negatively associated. Higher perceived responsibility at baseline was associated with a lesser change in saturated fat. Conversely, higher perceived fresh food availability was associated with a greater change in saturated fat. The baseline knowledge and attitudes model accounted for 12% of the variance in change in saturated fat (p=0.006) (Table 3).

Restriction (β=1.0, p=0.01) and pressure to eat (β=0.9, p=0.01) at baseline were positively associated with change in saturated fat intake (Model 3a). Higher restriction and pressure to eat were associated with a lesser change in saturated fat intake over the course of the intervention. The baseline parent shaping practices model (Model 3a) accounted for 16% of the variance in change in saturated fat intake (p<0.001) (Table 3). From the parent behaviour and role modelling model (Model 4a), only family involvement in meal preparations (β=1.2, p=0.03) was independently (and positively) associated with change in saturated fat (Table 3).

The regression analysis for change in family food environment and change in percentage energy from saturated fat is presented in Table 4. The change in knowledge and attitudes variables accounted for 18% of the variance in the change in children’s saturated fat intake (Model 2b, p<0.001). There were inverse associations observed between both change in dairy message (β=-0.8, p=0.01) and general nutrition knowledge (β=-0.1, p=0.03) with degree of change in saturated fat. With increasing knowledge the decrease in percent energy from saturated fat was greater. An increased perceived responsibility for children’s food intake (β=-1.4, p<0.001) was also inversely associated with change in saturated fat (Model 2b, Table 4).

**Table 4:** Results for multiple linear hierarchical regression for change in family food environment and change in percentage of energy from saturated fat

	**β**^**1**^	**Std β**	**95% CI**		**P value**	**Adjusted R**^**2**^	**Model P value**
Model 2b^3^ Knowledge and attitudes						0.18	<0.001
Dairy messages (/3)	-0.8	-0.2	-1.3	-0.2	0.01		
Nutrition knowledge (/113)	-0.1	-0.2	-0.2	-0.01	0.03		
Perceived responsibility (/5)	-1.4	-0.3	-2.3	-0.4	<0.001		
Perceived fresh food availability (/5)	0.3	0.05	-0.7	1.4	0.60		
Perceived adequacy of child's diet (/5)	-0.7	-0.1	-1.8	0.5	0.20		
Parent’s meal preparation views (/5)	-0.9	-0.1	-2.1	0.3	0.10		
Concern for weight (/5)	0.4	0.1	-0.4	1.2	0.30		
Model 3b^3^ Parent shaping practices						0.11	0.006
Teaching encouraging child about food (/5)	-0.5	-0.05	-2.3	1.3	0.60		
Restriction (/5)	-1.4	-0.3	-2.3	-0.6	<0.001		
Pressure to eat (/5)	-0.3	-0.1	-1.0	0.5	0.50		
Monitoring (/5)	0.2	0.03	-0.8	1.2	0.70		
Model 4b^3^ Parent behaviours and role modelling						0.04	0.136
Food involvement (/7)	-0.4	-0.1	-1.5	0.7	0.50		
Family involvement in meal preparation (/5)	1.0	0.1	-0.5	2.6	0.20		
Role modelling eating behaviours (/5)	-0.4	-0.04	-2.1	1.4	0.70		
TV interruptions to meals (/5)	0.1	0.02	-0.9	1.1	0.90		

^1^ Negative Beta value can be interpreted as an increase in the food environment construct was associated with a greater decrease in saturated fat. A positive Beta value can be interpreted as an decrease in the food environment construct was associated with a greater decrease in saturated fat.

^2^ Highest level of education completed (4 categories: primary school or less, some/completed high school, vocational/trade qualification, tertiary degree) and total household income (4 categories) were treated as continuous variables.

^3^ Analyses account for child (gender, age and BMI z score) and family demographics (household income, parent education and BMI).

Greater increase in restriction was associated with a greater decrease in saturated fat (β=-1.4, p<0.001, Model 3b, Table 4). The change in the parent shaping practices model accounted for 11% of variance in the change in saturated fat intake (p=0.006). Role modelling factors were not associated with change in saturated fat intake (Model 4b, Table 4).

## Discussion

This study examined whether baseline and change in characteristics of the family food environment were associated with change in children’s diet, specifically saturated fat intake. The present study is one of the first to measure changes in the family food environment as simultaneous correlates of dietary change in children. Some measureable changes in these variables were observed, and change in parents’ knowledge, perceived responsibility and restriction were associated with positive dietary change in children. The key finding of this study was that factors associated with change in saturated fat intake differed depending on whether baseline or change in the family food environment was the exposure of interest.

A number of family food environment factors, measured at baseline, were associated with a change in children’s saturated fat intake. Higher parent reported perceived responsibility for food provision, greater perceived food availability, lower levels of restriction and pressure to eat were associated with greater decrease in saturated fat intake over the 12 week intervention period. Such findings support previous cross-sectional studies where child feeding practices, such as restriction and higher pressure to eat, have been associated with less healthy behaviours [6]. Cross-sectional research has also shown that television interruptions to meals, parent’s role modelling and perceptions of adequacy of their child’s diet are important influences of children’s current dietary behaviour [6], and parents teaching their children about nutrition has been found to be associated with children’s current weight status [38]. However, the present study found these variables were not associated with a change in diet. This may reflect limited variation in the data or a distinction between influences of current versus change in behaviour. Indentifying who is most likely to respond to an intervention based on demographic or other baseline characteristics, may increase the likelihood of successful behaviour change. Understanding and focusing on the determinants of change in the family food environment may also improve the effectiveness of intervention strategies to improve children’s diet and health outcomes.

The present study examined how changes in the family food environment were associated with changes in intake. Increases in nutrition knowledge, perceived responsibility and restriction were associated with a decrease in children’s saturated fat intake. The regression models suggest that parents’ knowledge and attitudes and shaping practices explain a greater proportion of variance in the change in children’s diet than role modelling aspects of the family environment. It has been shown that role modelling is an essential part of adopting new behaviour [39], and children tend to model their parents’ eating behaviours [40], which is evident from research showing parent and children’s intakes of nutrients and food groups are generally correlated [14,15]. Longitudinal research with Australian adolescents has shown baseline levels of role modelling of healthy eating by mothers was associated with change in fruit consumption [19]. However, to the best of our knowledge, the present study is the first to capture changes in parent reported perceived role modelling as a result of intervention. The lack of significant findings further support the idea that predictors of current and change in behaviour may be different. Other possible explanations may be that role modelling specific to dairy foods may not translate in a more general food related role modelling questionnaire, or despite using validated questionnaires, it is difficult for questionnaires to detect such small changes. Also, parents may not be very aware of their own behaviours and realise the extent to which their behaviours influence the behaviour of their children. To highlight the importance of role modelling as a predictor of dietary change, and support its inclusion in interventions, it is necessary that questionnaires are well developed to measure this complex domain.

Knowledge is the foundation of nutrition education. Many interventions include nutrition education as part of their content, however, there have been few explorations of whether change in knowledge is associated with change in dietary behaviour. An increase in parents’ nutrition knowledge was associated with a decrease in children’s saturated fat intake. However, we know that knowledge is “required but not sufficient” for changes in food behaviour [41]. Improving knowledge is an attractive target of intervention because it is a relatively malleable characteristic, and at a population level knowledge of individuals is most amendable to policy intervention [42]. The results of this study support the inclusion of nutrition knowledge as an intervention target, in combination with other known determinants of behaviour change.

The multivariable regression analysis in the present study found differing results for baseline levels of restriction and change in restriction as a predictor of dietary change. *Lower* levels of parent reported restriction at baseline but an *increase* in restriction over the intervention period were associated with a decrease in saturated fat, or healthier behaviour. Previous parent-child feeding research has focussed on parental restriction and subsequent impacts on children’s energy intake and risk of obesity [9,43]. Higher levels of restriction and excessive control by parents can result in a reduction in children’s ability to self-regulate their own intake, resulting in higher energy intake [9,43]. In terms of energy balance, it appears higher levels of restriction are associated with negative eating behaviours in children. But in terms of food intake, one study to examine feeding practices and children’s consumption of certain food groups found no relationship between parental restriction and intake of healthy or unhealthy foods [6]. Therefore, there is still uncertainty as to the role of parental restriction on children’s eating behaviours. A review of parent-child feeding practices suggests the short and longer-term effect of shaping practices on children’s eating behaviours may be different [44]. From the results of this study, it is difficult to make inferences about the effects of restriction in promoting healthy dietary intake. The CFQ asks about restriction related to consumption of sweet and snack foods, junk foods and children’s favourite foods [10]. While the intervention focussed on decreasing the availability of regular fat milk at home, it is possible that parents perceived this behaviour to be a form of restrictive practice, thereby recording an increase in their response to these questions. Nonetheless, there appears to be a fine line between positive and negative impacts of restrictive practices and children’s eating behaviours [45], particularly in terms of healthy behaviours, and further research is required to decipher how parents’ shaping practices are best included into future interventions.

The family food environment factors showed acceptable validity. It is difficult to capture the complexity of the family environment through a questionnaire, however, using a comprehensive measure of known validity, may give greater confidence in these findings. Generally relatively small effect sizes were observed for changes in any one food environment factor. There may be a number of possible explanations for this. While the intervention aimed to change the food environment through food choices, support from parents and role modelling, it is possible that it was not successful in changing these domains, or that scores at baseline were high and therefore results showed evidence of a ceiling effect. But it is also possible that the intervention was successful in changing these, however, the tools used did not capture this change because these tools have generally been developed within obesity literature. Regardless, we were still able to capture a range of changes in the family food environment. Because understanding change in family food environments is vital to intervention success, further effort is needed to examine the sensitivity of existing tools to detect change or develop new tools if required.

In considering these findings, it is important to acknowledge some of the limitations of this study. While parent and children’s sociodemographic characteristics and weight status largely represents that of broader Australian community [36,46,47], this sample was relatively small and was comprised of volunteers who may show greater motivation than the general population. As a result, baseline and change in the family food environment and dietary intake of children may be more positive than what could be expected generally. All predictor variables of the family food environment were theoretically derived but were self-reported, and are subject to social desirability bias. A strength of this study is the rigorous dietary assessment method used. Three 24-hour recalls is considered a robust measure of diet in children [48]. Parent report is recommended for younger children, but because this sample contained children aged 4-13 years, the primary source of reporting was not consistent between the younger and older children. Another strength was that the family food environment was measured pre and post intervention, allowing change to be calculated. Other multifaceted studies have had limited scope to capture the complexity of the family environment. So a further strength of this study is the detail to which the food environment was measured, and the broad range of family characteristics that were measured. While we attempted to capture the complexity of the family food environment, it is acknowledge that there are numerous other factors that can influence behaviour change which should also be considered in future research. For example children’s personal characteristics such as self-esteem or factors from within the community or wider environment. The influences of behaviour may vary with children’s age. Future research may examine the change in the relative importance of the family and other environments as young children transition through to adolescents.

## Conclusion

The family food environment inevitably plays an important role in the development of children’s dietary habits [49]. The present study was able to quantify changes in the family food environment and identify a number of factors which were associated with a decrease in children’s saturated fat intake. Nutrition interventions target behaviour change as opposed to current behaviour, therefore the findings of this research may provide specific target for intervention strategies in the future.

## Competing interests

The research was supported by CSIRO Food and Nutrition Sciences. GS was a Flinders University Nutrition and Dietetics Masters Student. RKG is funded by a NHMRC public health training award (478115). The RCT was funded by Dairy Australia. The study was conducted and this manuscript prepared without input from Dairy Australia (the funding body). Dairy Australia approved this manuscript for publication. All authors declare no conflicts of interest.

## Authors’ contributions

GH and RG were responsible for the design and completion of the RCT. GH and RG conceived this study. KL provided statistical expertise, assisted with the data analysis and interpretation. RG conducted the analysis. GH drafted the manuscript. GS assisted with the data analysis and draft of the manuscript. All authors read and approved the final manuscript.
